# Bacterial synthesis of anisotropic gold nanoparticles

**DOI:** 10.1007/s00253-025-13438-w

**Published:** 2025-03-10

**Authors:** Islam M. Ahmady, Javad B. M. Parambath, Elsiddig A. E. Elsheikh, Gwangmin Kim, Changseok Han, Alejandro Pérez-García, Ahmed A. Mohamed

**Affiliations:** 1 Department of Applied Biology, College of Sciences, University of Sharjah, 27272 Sharjah, United Arab Emirates; 2 Departamento de Microbiología, Universidad de Málaga, and Instituto de Hortofruticultura Subtropical y Mediterránea “La Mayora” (IHSM-UMA-CSIC), 29071 Málaga, Spain; 3 Center for Advanced Materials Research, Research Institute of Sciences and Engineering, University of Sharjah, 27272 Sharjah, United Arab Emirates; 4 Program in Environmental and Polymer Engineering, Graduate School of INHA University, 100 Inha-Ro, Michuhol-Gu, Incheon, 22212 Korea; 5 Department of Environmental Engineering, INHA University, 100 Inha-Ro, Michuhol-Gu, Incheon, 22212 Korea

**Keywords:** Anisotropic gold nanoparticles, Aryldiazonium gold(III), Bacterial metabolites, *Pseudomonas aeruginosa*

## Abstract

**Abstract:**

*Pseudomonas aeruginosa *was used to synthesize anisotropic gold nanoparticles from the unusually reducible aryldiazonium gold (III) salt of the chemical formula [HOOC-4-C_6_H_4_N≡N]AuCl_4_ (abbreviated as DS-AuCl_4_). We investigated the effect of bacterial cell density, temperature, and pH on the AuNP synthesis. The bacterial cell density of 6.0 × 10^8^ CFU/mL successfully reduced 0.5 mM DS-AuCl_4_ salt to AuNPs after incubation at 37 °C (24 h), 42 °C (24 h), and 25 °C (48 h). Transmission electron microscopy (TEM) images revealed the formation of spherical, triangle, star, hexagon, and truncated triangular morphologies for the AuNPs synthesized using *P. aeruginosa* bacteria. The average size of AuNPs synthesized at 25 °C (48 h), 37 °C (24 h), and 42 °C (24 h) was 39.0 ± 9.1 nm, 26.0 ± 8.1 nm, and 36.7 ± 7.7 nm, respectively. The average size of AuNPs synthesized at pH 3.7, 7.0, and 12.7 was 36.7 ± 7.7 nm, 14.7 ± 3.8 nm, and 7.3 ± 2.5 nm, respectively, with the average size decreasing at a pH of 12.7. The reduction of the DS-AuCl_4_ salt was confirmed using X-ray photoelectron spectroscopy (XPS). The significant peaks for C1s, Au4f doublet, N1s, and O1s are centered at 285, 84–88, 400, and 532 eV. The ability of inactivated bacteria (autoclave-dead and mechanically lysed bacteria), peptidoglycan, and lipopolysaccharides to reduce the DS-AuCl_4_ salt to AuNPs was also investigated. Anisotropic AuNPs were synthesized using inactivated bacteria and peptidoglycan but not using lipopolysaccharides. The AuNPs demonstrated biocompatibility with human RBCs and were safe, with no antibacterial activities against *Escherichia coli* and *Staphylococcus aureus*. This is the first report demonstrating the synthesis of AuNPs using aryldiazonium gold(III) salts with *P. aeruginosa*. These AuNPs are promising candidates for exploring potential applications in nanomedicine and drug delivery.

**Key points:**

• *Anisotropic AuNPs were synthesized using P. aeruginosa bacteria.*

• *Dead and lysed bacterial residues synthesized anisotropic AuNPs.*

• *AuNPs are hemocompatible.*

**Graphical Abstract:**

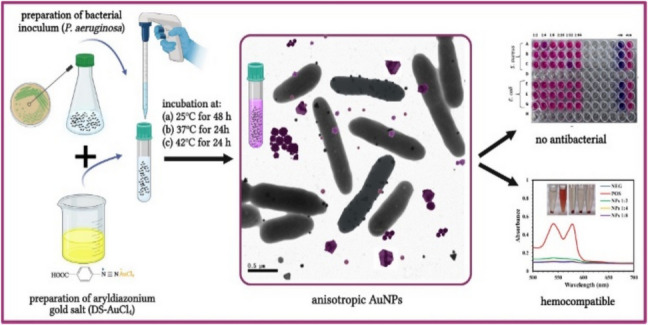

**Supplementary Information:**

The online version contains supplementary material available at 10.1007/s00253-025-13438-w.

## Introduction

Gold nanoparticles are the most favored choice in biomedical applications due to their low toxicity (Butler et al. 2023). However, their synthetic methods that involve toxic chemical reagents can limit their in vivo applications. Therefore, developing eco-friendly procedures for biocompatible AuNP synthesis is crucial. Green synthesis routes that use biological reducing agents, including microorganisms, enzymes, sugars, secondary metabolites, and plant extracts, have provided promising approaches for biocompatible AuNP fabrication (Marslin et al. 2018; Hameed et al. 2020; Jayeoye et al. 2024; Karnwal et al. 2024). Moreover, it is crucial to synthesize water-soluble AuNPs that can withstand physiological conditions. Gold-aryl NPs utilize highly biocompatible and water-soluble organic shell coating. Our research has successfully used proteins and amino acids for AuNP synthesis from water-soluble aryldiazonium gold(III) salts (DS-AuCl_4_) for biomedical applications. The biocompatible gold-aryl NPs synthesized using lysozyme protein showed remarkable antibacterial activities against multidrug-resistant bacteria, including the extended-spectrum β-lactamases (ESBL)-producing *E. coli* and imipenem-resistant *P. aeruginosa* (Ahmady et al. 2019). Research groups have investigated DS-AuCl_4_ salts because they are easily reducible using chemical reducing agents, electrochemical processes, irradiation stimuli, and spontaneously without chemical reducing agents. A galvanic replacement reaction was used to synthesize gold-silver alloy nanoparticles. The alloy nanoparticles showed antibacterial properties against *S. aureus*, *S. epidermidis*, *E. faecalis*, *E. coli*, *S. typhimurium*, and *P. aeruginosa* (Parambath et al. 2022). Gold-silver nanoparticles were synthesized using the DS-AuCl_4_ salt to investigate the effect of ionic silver release on the antibacterial activity (Panicker et al. 2020).

Anisotropic AuNPs offer distinct advantages, including tunable plasmonic properties, enhanced surface area, anisotropic surface chemistry, and morphology-dependent optical properties, which make them highly desirable for biomedicine, optics, and catalysis applications (Montaño-Priede et al. 2024; Ortiz-Castillo et al. 2020; Xia et al. 2023). Anisotropic AuNP synthesis poses significant challenges, which are more pronounced in microbial synthesis, as controlling the growth parameters is not straightforward (Ortiz-Castillo et al. 2020). There is a high interest in investigating a facile, eco-friendly process to synthesize robust anisotropic AuNPs. Two morphologies were fabricated based on the DS-AuCl_4_ salts: nanostars (Hameed et al. 2022) and cubic (Parambath et al. 2023a, b). Arylated gold nanostars were synthesized using the seeding growth method, which was used to detect breast cancer cells using surface-enhanced Raman spectroscopy (SERS). We synthesized cubic-shaped gold nanoparticles based on the DS-AuCl_4_ salts, which were used to monitor the organic pollutants using SERS (Parambath et al. 2023a, b).

Microorganisms can produce NPs of varying morphologies using intracellularly and extracellularly mechanisms by utilizing chemical detoxification and energy-dependent ion efflux mechanisms through membrane proteins functioning as ATPase, chemiosmotic cation, or antiproton transporters to resist toxic metals (Ghosh et al. 2021; Park et al. 2016). Various bacterial strains can produce metal NPs with different morphologies (Carmona et al. 2023; Khan et al. 2024; Verma et al. 2024). The reduction capability can be due to bacterial cells containing reducing carbohydrates and proteins (Khandel and Shah 2016; Park et al. 2016). *P. aeruginosa*, Gram-negative bacteria, thrives in soil and wet environments and can endure temperatures as high as 42 °C while exhibiting slow growth at 25 °C. *P. aeruginosa* can produce NPs with different morphologies from Au via intracellular and extracellular mechanisms (Singh et al. 2013). The reduction of HAuCl_4_ using *P. aeruginosa* supernatant and the formation of spherical AuNPs has been previously reported (Husseiny et al. 2007).

In this study, we (1) assessed the optimal conditions for *P. aeruginosa* to synthesize anisotropic AuNPs under different bacterial cell density, temperature, and pH, (2) investigated the ability of autoclave-dead and mechanically lysed bacterial cells to synthesize AuNPs, (3) assessed the ability of peptidoglycan and lipopolysaccharides to reduce the DS-AuCl_4_ salt at different concentrations and synthesize AuNPs, (4) evaluated the synthesis of AuNPs using bacterial metabolites after different time intervals of incubation, and (5) assessed AuNP antibacterial activity and hemocompatibility to human blood cells. To the best of our knowledge, this is the first report showing the ability of microorganisms to synthesize AuNPs using the easily reducible DS-AuCl_4_ salts (Abdou et al. 2007; Chen et al. 1999).

## Material and methods

### Synthesis of [HOOC-4-C_6_H_4_N≡N]AuCl_4_ salt

Chloroauric acid (HAuCl_4_) was synthesized from 99.9999% gold metal by dissolving gold in aqua regia followed by evaporation. Water-soluble aryldiazonium gold(III) salt [HOOC-4-C_6_H_4_N≡N]AuCl_4_, abbreviated in the following texts as DS-AuCl_4_, was synthesized using the method outlined previously (Ahmad et al. 2019). Briefly, 5 mL of a 4.5 mM aqueous solution of NaNO_2_ was added dropwise to a solution of 15 mL of 3 mM 4-aminobenzoic acid dissolved in 6 M HCl while continuously stirring for 1 h in an ice bath. Then, 10 mL of 3 mM aqueous solution of HAuCl_4_·3H_2_O was added slowly under vigorous stirring in an ice bath until a canary yellow microcrystalline precipitate formed. The residue was washed with cold water, dried, and kept in the fridge.

### Bacterial strains and growth conditions

*Pseudomonas aeruginosa* ATCC 27853, *Escherichia coli* ATCC 25922, and *Staphylococcus aureus* ATCC 29213 were obtained from the American Type Culture Collection (ATCC). Bacterial strains were cultured on nutrient agar and incubated at 37 °C overnight.

### Blood samples

Blood samples (O positive) were collected from a healthy donor. Following the declaration of Helsinki Guidelines, the Ethics Committee at the University of Sharjah, REC-22–12-03–01-Other, approved the blood experiments. The donor gave informed consent for the study.

### Experimental design

#### Preparation of bacterial inoculum

Liquid cultures of *P. aeruginosa* were grown overnight on nutrient broth and then centrifuged at 5000 rpm for 10 min. The broth was discarded, replaced with 25 mL of fresh broth, and allowed to grow for 2–3 h on a shaking incubator at 37 °C to reach the log phase. Then, the bacteria were centrifuged and washed 3 × with deionized water to remove the metabolites and broth.

#### Effect of bacterial cell density and temperature on AuNP synthesis

Three bacterial cell concentrations were assessed: 1.5 × 10^8^, 3.0 × 10^8^, and 6.0 × 10^8^ CFU/mL, equal to 0.5, 1.0, and 2.0 McFarland turbidity standard solution. The inoculum OD was adjusted to the corresponding McFarland concentration at 600 nm using a Densichek turbidity meter (bioMérieux, Marcy-l'Étoile, France). Synthesis of AuNPs was evaluated at 25 °C, 37 °C, and 42 °C using 0.5 mM DS-AuCl_4_. Equal volumes of 2-mL bacteria and 2-mL DS-AuCl_4_ were mixed to achieve the targeted concentrations. A tube with DS-AuCl_4_ and deionized water without bacteria was used as a control. Then, samples were incubated aerobically at three temperatures in the dark undisturbed and monitored daily. The successful formation of AuNPs was visually observed by the change in color and was confirmed using a UV–vis microplate spectrophotometer BioTek Epoch 2 (Agilent Technologies, Santa Clara, CA, USA). The experiment was repeated three times to ensure the accuracy of the result.

#### Effect of pH on AuNP synthesis

To examine the effect of pH on the synthesis of AuNPs using *P. aeruginosa* bacteria, a cell density of 6.0 × 10^8^ CFU/mL and 0.5 mM DS-AuCl_4_ were used. Three different pH values were evaluated: 3.7 (original solution pH), 7.0, and 12.7. The pH of the DS-AuCl_4_ solution was adjusted using 0.1 M NaOH. The samples were then incubated aerobically at 42 °C in the dark and static conditions and monitored using UV–vis. The experiment was repeated three times to ensure the accuracy of the result.

#### Synthesis of AuNP using autoclave-dead and mechanically lysed bacterial cells

Cultures of *P. aeruginosa* were grown in 25 mL of fresh broth for 2–3 h on a shaking incubator at 37 °C. Then, the bacterial cultures were autoclaved and centrifuged at 5000 rpm for 10 min to collect the pellets of dead bacterial cells. The pellets were washed 3 × with deionized water. An inoculum equivalent to a cell density of 2.0 McFarland (6.0 × 10^8^ CFU/mL) was combined with 0.5 mM DS-AuCl_4_ and incubated aerobically at 25 °C, 37 °C, and 42 °C in the dark and static conditions. A control sample of DS-AuCl_4_ without bacterial cells was used, and samples were monitored using UV–vis. The experiment was repeated three times to ensure the accuracy of the result. To mechanically lyse the cells, *P. aeruginosa* was cultured in 20 mL of nutrient broth overnight, and then the pellets were collected and washed 3 × with deionized water. Next, five glass beads were added, and the tubes were vortexed for 15 min and sonicated for another 15 min. Then, the lysed bacterial cell residues were collected by centrifugation at 5000 rpm and washed 3 × with deionized water. The residues were diluted with deionized water to a final cell density of 2.0 McFarland and added to DS-AuCl_4_ at a final concentration of 0.5 mM. The samples were incubated at 42 °C and monitored using UV–vis. The experiment was repeated three times to ensure the accuracy of the result.

#### Synthesis of AuNPs using peptidoglycan and lipopolysaccharides

The examination was carried out to determine the ability of pure peptidoglycan of *Bacillus subtilis* (Sigma-Aldrich, Saint Louis, MO, USA) and lipopolysaccharides from *E. coli* (Sigma-Aldrich) to synthesize the AuNPs in the absence of bacterial cells. Stock solutions were prepared: (a) 10 mg of peptidoglycan was dissolved in 1 mL of deionized water, and (b) 10 mg and 200 mg of lipopolysaccharide separately were dissolved in 1 mL of deionized water. Aliquots of 200 µL, 100 µL, and 25 µL of each concentration were added into Eppendorf tubes containing 1 mL of 0.5 mM DS-AuCl_4_. The samples were incubated at 37 °C until a noticeable color change occurred.

#### Synthesis of AuNPs using extracellular metabolites

An experiment was designed to study the ability of extracellular metabolites from *P. aeruginosa* to synthesize AuNPs. The bacteria were grown in nutrient broth for four days at 37 °C. The supernatants containing the metabolites were collected after 24 h, 48 h, and 96 h and filtered through 0.22-µm filters to remove the bacterial cells. Then, 2 mL of 1.0 mM DS-AuCl_4_ was added to 2 mL of filtered supernatant samples containing metabolites to give a final concentration of 0.5 mM of DS-AuCl_4_. The samples were incubated at 42 °C and monitored using UV–vis. Two control samples were prepared: DS-AuCl_4_ and nutrient broth as a negative control and DS-AuCl_4_ and NaBH_4_ as a positive control. The experiment was repeated three times to ensure the accuracy of the result.

#### High-resolution transmission electron microscopy (HR-TEM), energy dispersive spectroscopy (EDS), and field emission scanning electron microscopy (FE-SEM)

HR-TEM and FE-SEM analyses were performed to investigate the physical properties and surface morphology of AuNPs. A JEM-2100F microscope (JEOL, Akishima, Japan) was used for HR-TEM measurements. The samples were immobilized on 200 mesh copper TEM grids with a carbon support film CF200-Cu-50 (Electron Microscopy Sciences, Hatfield, PA, USA). A few drops of the sample solutions were dropped on the TEM grids and then dried at room temperature. Then, samples were negatively stained using the non-radioactive contrast stain UranyLess (Electron Microscopy Sciences). The HR-TEM was equipped with an energy dispersive spectroscopy (EDS). The EDS was used to identify gold presence in the samples qualitatively. A S-4300SE microscope (Hitachi, Chiyoda, Japan) was used for FE-SEM measurements. The samples were dropped and dried at room temperature on 26 mm specimen stubs. The dried samples were coated with Pt using a Turbo Pumped Sputter Coater Q150T (Quorum, Laughton, UK) and subjected to SEM analysis.

#### X-ray powder diffraction (XRD) and X-ray photoelectron spectroscopy (XPS)

X-ray powder diffraction (XRD) was performed to identify the phase of the crystalline material. XRD data were collected using an X-ray diffraction platform D8 ADVANCE (Bruker, Billerica, MA, USA) with a Cu Kα X-ray source at λ = 1.5406 Å operating at 40 kV tube voltage and 40 mA current. To examine the elemental composition and chemical oxidation state, X-ray photoelectron spectroscopy (XPS) was conducted. For this purpose, a Nexsa G2 surface analysis system (Thermo Fisher Scientific, Waltham, MA, USA) was used. The instrument utilized monochromatized Al-Kα radiation (1486.6 eV) with a spot size of 400 μm and used a flood gun for static charge compensation. Survey spectra were acquired with a pass energy of 200 eV, while high-resolution scans were conducted at 50 eV, all under an ultra-high vacuum environment of 10^−8^ mbar.

#### Antibacterial activity of DS-AuCl_4_ salt and AuNPs

The inhibitory activity of DS-AuCl_4_ against *P. aeruginosa* ATCC 27853 strain was evaluated using the macrodilution (tube) broth method following the guidelines of the Clinical and Laboratory Standards Institute (CLSI) (Weinstein et al. 2018). The procedure involved 1 mL of twofold decreasing concentrations ranging from 4.0 mM to 0.016 mM DS-AuCl_4_ diluted in Mueller–Hinton broth. Then, *P. aeruginosa* was added at a final concentration of 5 × 10^5^ CFU/mL, and the test tubes were incubated overnight at 37 °C. Positive (with bacteria) and negative (without bacteria) controls were prepared. The sample was run in triplicate. Growth was acceptable if turbidity was observed in the test tubes. The final tube that showed no visible growth was recorded as the minimum inhibitory concentration (MIC). Based on the inhibitory activity test, it was found that the MIC of DS-AuCl_4_ was 0.125 mM.

The antibacterial activity of purified AuNPs synthesized using live bacteria and metabolites, collected after 96 h and incubated at 42 °C, was assessed against Gram-negative *E. coli* ATCC 25922 and Gram-positive *S. aureus* ATCC 29213 using the broth microdilution method in 96-micro-titer plates (Ahmady et al. 2019; Weinstein et al. 2018). Briefly, AuNPs were diluted twofold in Muller Hinton broth, then 0.01 mL of bacteria was added at a count of 5 × 10^5^ CFU/mL, and samples were run in triplicate. Both positive and negative control wells were prepared. Plates were sealed and incubated overnight, and shaken at 37 °C. The presence of ≥ 2 mm button or definite turbidity is an acceptable growth. The inhibitory activity was confirmed by adding a blue resazurin sodium reagent (Sigma-Aldrich) (Elshikh et al. 2016; Panicker et al. 2020). AuNPs were purified by filtering out the bacterial cells with 0.22 µm filters, followed by dialysis using a 25 mm tubing cellulose membrane with a 14,000 Da molecular weight cut-off (Sigma-Aldrich) to eliminate unreacted ions.

#### Hemocompatibility assay

The hemocompatibility assay was conducted using an O-positive blood sample collected in ethylenediamine tetraacetic acid (EDTA) from a healthy donor. The percentage of hemolysis and quantitative estimation of leached-out hemoglobin and red blood cells (RBCs) morphological changes were detected following the protocol established by previous studies (Ahmady et al. 2019; Zhao et al. 2011). Packed RBCs were separated from plasma by centrifugation at 1500 rpm for 5 min and washed 3 × with normal saline. Then, 200 µL of 5% RBCs were added to 800 µL of purified AuNPs diluted to 1:2, 1:4, and 1:8 in normal saline. Samples were incubated overnight in a shaking water bath at 37 °C. Negative (5% RBCs with normal saline) and positive control (5% RBCs with deionized water) were prepared. Then, samples of 100 µL were transferred into a 96-well plate, and absorbance was measured in the 450–800-nm range to quantify the leach-out hemoglobin. Hemolysis was evaluated after 1 h and 24 h, and the percentage of hemolysis was determined at 541 nm using the following formula, where OD is the optical density:

$$\mathrm{Hemolysis}(\mathbf{\%})= \frac{\text{sample OD }-\text{ negative control OD}}{\text{positive control OD }-\text{ negative control OD}} \times 100$$

The impact of AuNPs on the morphology of RBCs was investigated using phase contrast microscopy. In brief, tubes containing the treatments were shaken gently, and after 1 h and 24 h of incubation, 20 µL samples were transferred to a microscope slide and covered with a coverslip. RBCs images were taken using an inverted light and phase contrast microscope IX53 (Olympus, Shinjuku, Japan), equipped with a camera model DP74 (Olympus) of 1920 × 1200 pixels. The hemolysis assay experiments and the images showing the morphological changes in RBCs were studied in triplicates.

#### Statistical analysis

Hemolysis percentages were analyzed for significant differences using the student’s t-test with GraphPad Prism version 10.2.3 for Windows (GraphPad Software, Boston, Massachusetts USA, www.graphpad.com). Statistically significant differences from the control group are indicated in the figures with ****, representing *p*-values < 0.0001. Error bars represent the ± standard errors from three independent replicates (*n* = 3).

## Results

### Synthesis and characterization of AuNPs produced using P. aeruginosa ATCC 27853

**Fig. 1:**
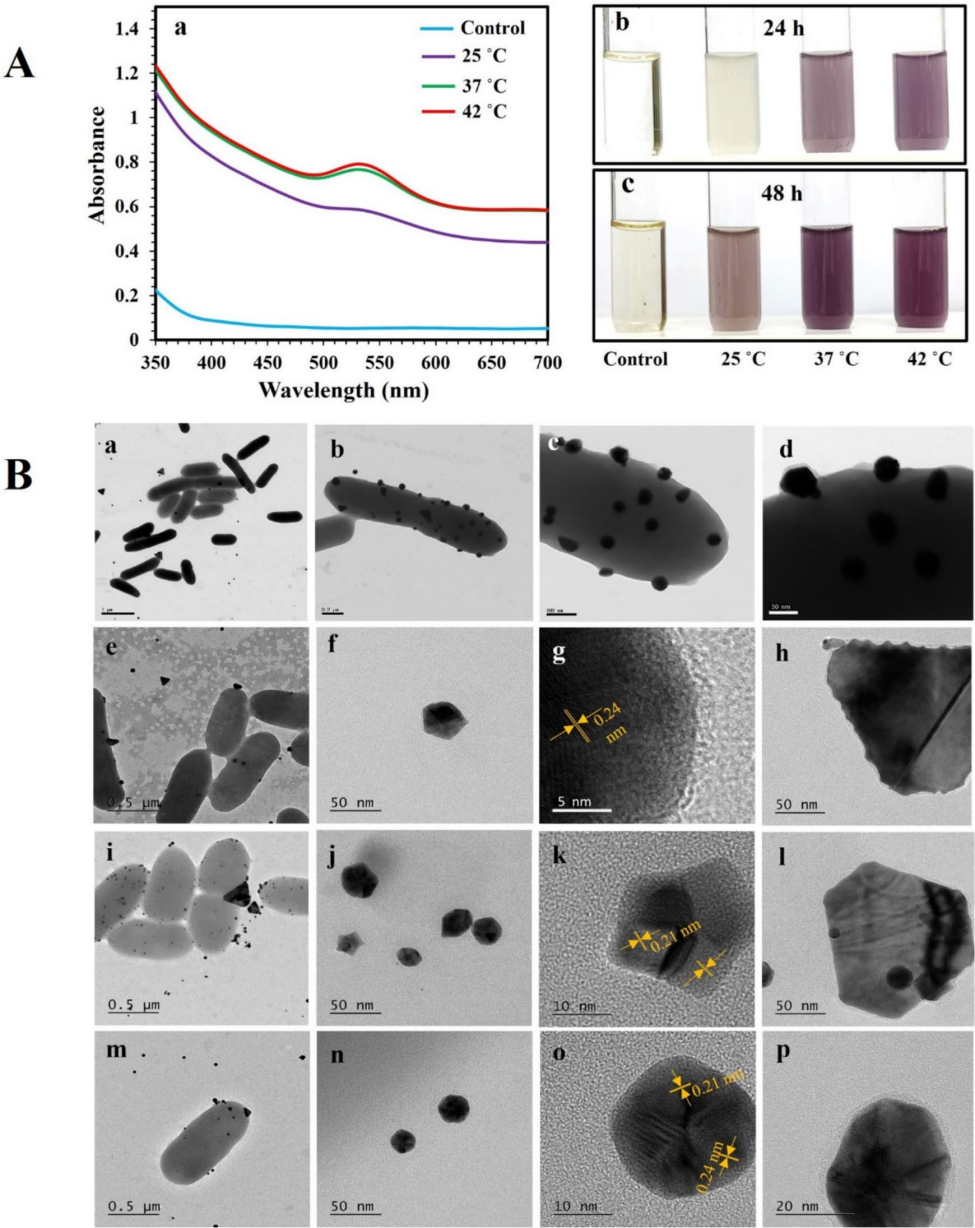
UV–vis and TEM investigation of AuNPs synthesized using *P. aeruginosa* ATCC 27853. Bacterial cell suspensions of 6.0 × 10^8^ CFU/mL were incubated with 0.5 mM DS-AuCl_4_ at 25 °C (48 h), 37 °C (24 h), and 42 °C (24 h). (**A**) Progression of the AuNPs. UV–vis was recorded after 48 h of incubation (a), and images of the test tubes display the progression of purple color after 24 and 48 h (b–c). (**B**) HR-TEM images of synthesized AuNPs at 25 °C (48 h) (a–h), 37 °C (24 h) (i–l), and 42 °C (24 h) (m–p). Lattice spacing of AuNPs is shown in orange. The experiments were repeated at least three times. Representative images of UV–vis, test tubes, and HR-TEM images are shown

The ability of *P. aeruginosa* to synthesize AuNPs was investigated at three bacterial cell densities of 1.5 × 10^8^, 3 × 10^8^, and 6 × 10^8^ cells/mL and temperatures of 25 °C, 37 °C, and 42 °C, using actively growing bacteria incubated with 0.5 mM DS-AuCl_4_. Figure 1A shows that a bacterial cell concentration of 6 × 10^8^ cells/mL successfully reduced DS-AuCl_4_ to AuNPs, whereas concentrations of 1.5 × 10^8^ and 3 × 10^8^ cells/mL were ineffective (Fig. S1). A purple color was formed, indicating the ability of *P. aeruginosa* to synthesize AuNPs. UV–vis was used to evaluate the synthesized AuNPs, and the plasmon peak was observed at 530 nm. When the samples were incubated at 25 °C, AuNPs formed after 48 h, while at 37 °C and 42 °C, they formed after 24 h. AuNPs were characterized using HR-TEM (Fig. 1B). AuNPs were observed around bacteria as spherical, triangular, star, hexagonal, and truncated triangles. The average size of AuNPs synthesized at 25 °C (48 h), 37 °C (24 h), and 42 °C (24 h) was 39.0 ± 9.1 nm, 26.0 ± 8.2 nm, and 36.7 ± 7.7 nm, respectively. The measured lattice spacing for all AuNPs was 0.21 nm and 0.24 nm, corresponding to (200) and (111) planes of Au. EDS analysis supported the presence of AuNPs.

### Effect of pH on AuNP synthesis

**Fig. 2:**
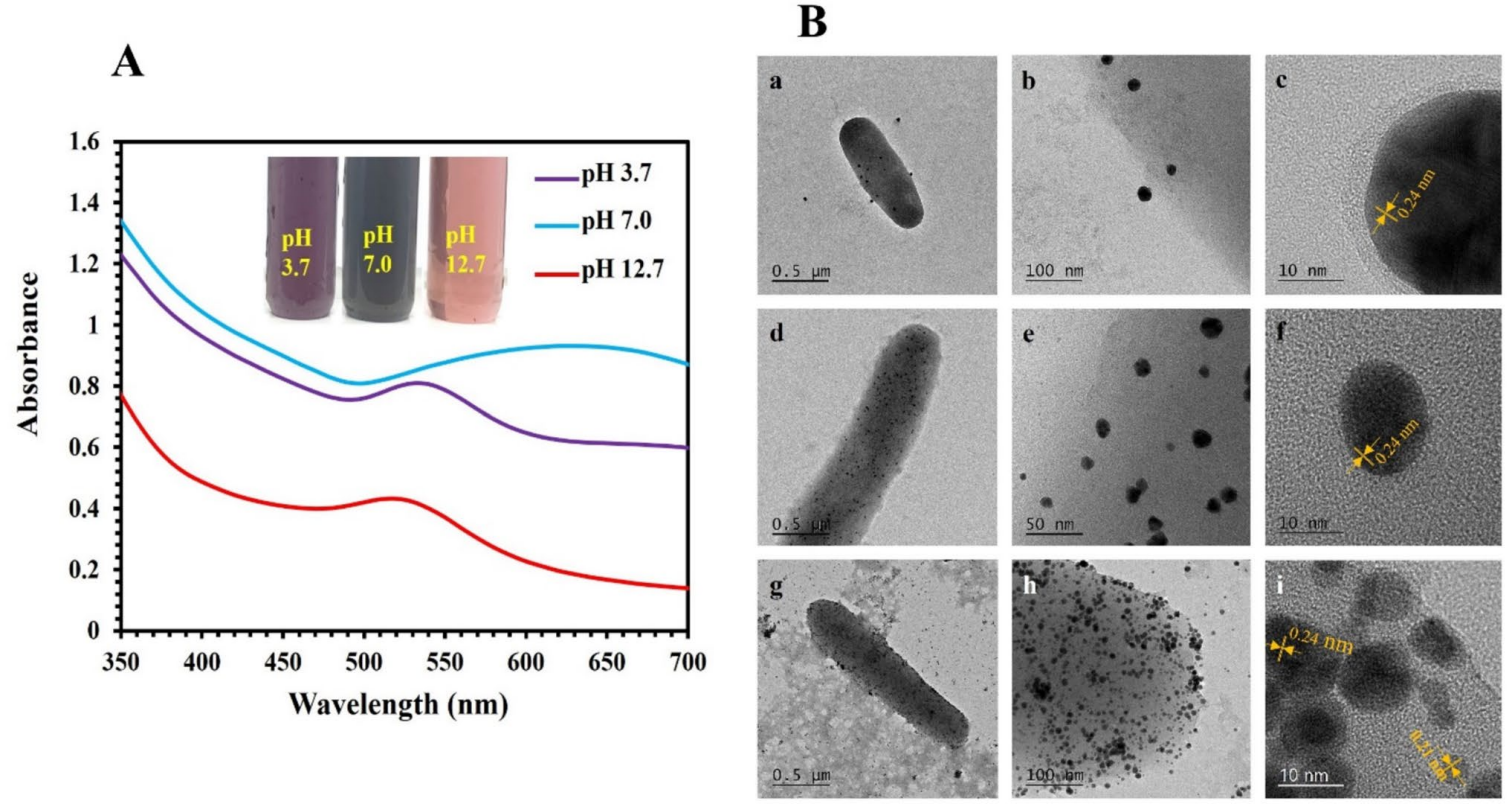
UV–vis and TEM investigation of the effect of pH on the synthesis of AuNPs using *P. aerug*inosa ATCC 27853. Bacterial cell suspensions of 6.0 × 10^8^ CFU/mL were incubated with 0.5 mM DS-AuCl_4_ at pH of 3.7, 7.0, 12.7, and 42 °C (24 h). (**A**) UV–vis after incubation and images of test tubes displaying the progression of AuNP colors. (**B**) HR-TEM images of synthesized AuNPs at pH 3.7 (a–c), pH 7.0 (d–f), and pH 12.7 (g–i). Lattice spacing of AuNPs is shown in orange. The experiments were repeated at least three times. Representative images of UV–vis, test tubes, and HR-TEM images are shown

The effect of the pH on the AuNP synthesis was evaluated at pH 3.7, 7.0, and 12.7, using a bacterial density of 6 × 10^8^ CFU/mL incubated at 42 °C. As shown in Fig. 2A, it was observed that different colors were produced at varying pH values in the test tubes. Purple, blue, and red colors were developed at pH 3.7, 7.0, and 12.7, respectively. UV–vis showed the plasmon peak at 530 nm at pH 3.7 and 12.7, while a broad peak centered at 640 nm was observed at pH 7.0. The blue shift from 530 to 520 nm suggested a decrease in particle size, as shown by the progression of the colors in the test tubes (Fig. 2A). HR-TEM analysis showed that the synthesized AuNPs using bacteria were different in size at different pH values (Fig. 2B). The average sizes of AuNPs synthesized at pH 3.7, 7.0, and 12.7 were 36.7 ± 7.7 nm, 14.7 ± 3.8 nm, and 7.3 ± 2.5 nm, respectively. The average sizes became smaller at a pH of 12.7. The measured lattice spacings of AuNPs were 0.21 nm and 0.24 nm, corresponding to (200) and (111) planes of Au. EDS analysis confirmed the presence of Au in the AuNPs.

### Synthesis of AuNPs using autoclave-dead and mechanically lysed bacterial cells

**Fig. 3:**
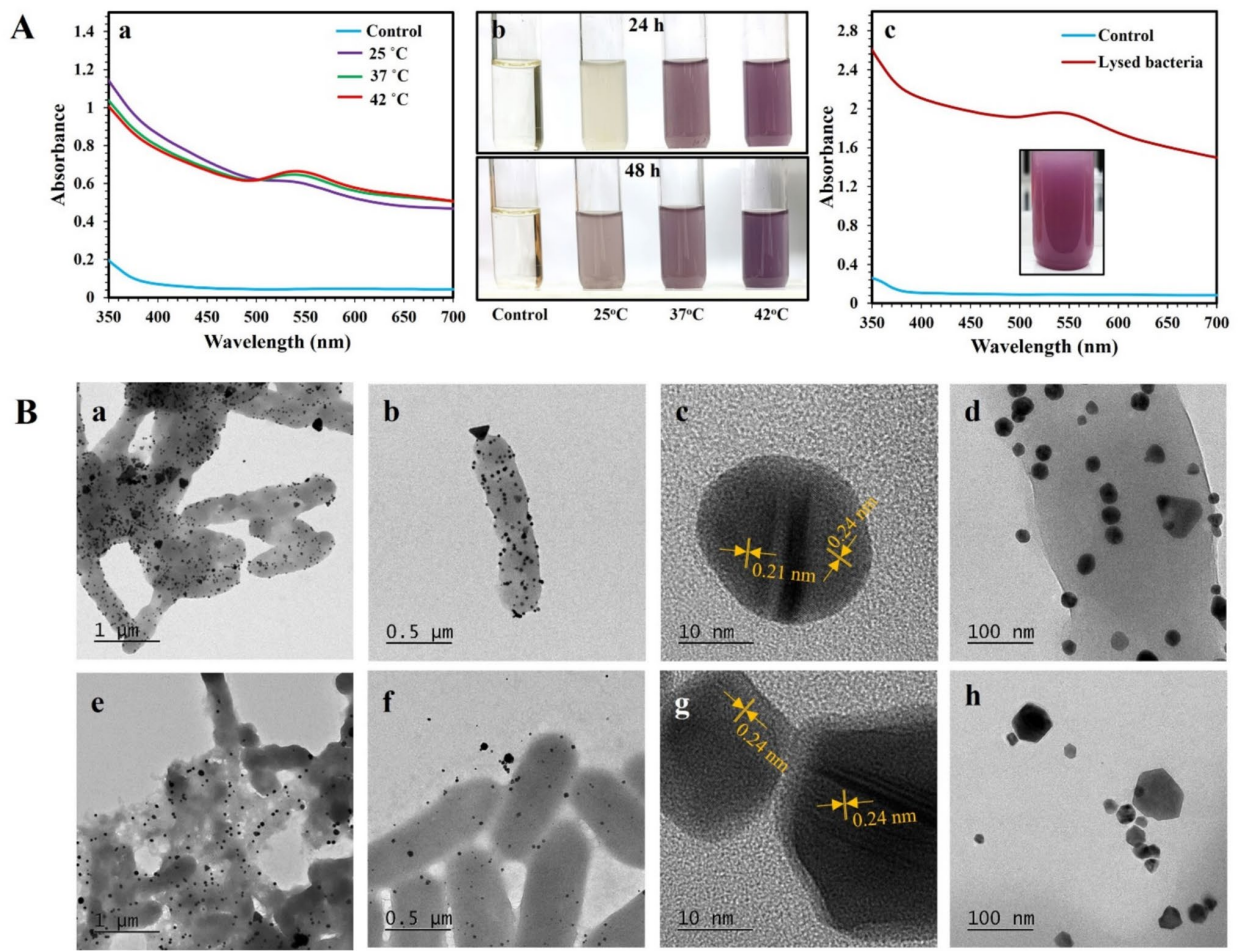
UV–vis and TEM investigation of AuNPs synthesized using autoclave-dead and mechanically lysed bacterial cells. Inactivated bacteria from 6.0 × 10^8^ CFU/mL cell suspensions were incubated with 0.5 mM of DS-AuCl_4_ at different temperatures. (**A**) UV–vis and tube tests. UV–vis of autoclave-dead cells recorded after 48 h of incubation (a) and images of the test tubes displaying the progression of purple color after 24 and 48 h of incubation (b). UV–vis of mechanically lysed bacteria incubated at 42 °C for 96 h and an image of the tube showing AuNP color change (c). (**B**) HR-TEM images of AuNPs synthesized using autoclave-dead bacteria at 42 °C (a–d) and mechanically lysed bacteria at 42 °C (e–h). Lattice spacing of AuNPs is shown in orange. The experiments were repeated at least three times. Representative images of UV–vis, test tubes, and HR-TEM images are shown

To test whether inactivated bacteria were able to reduce the gold salt and produce nanoparticles, experiments were conducted using autoclave-dead or mechanically lysed *P. aeruginosa* cells at a cell density of 6.0 × 10^8^ CFU/mL and 0.5 mM DS-AuCl_4_ (Fig. 3). For autoclave-dead cells, the purple color was developed after 48 h (25 °C) and 24 h (at 37 °C and 42 °C) (Fig. 3A), with an intensity similar to that of live bacteria. Similarly, mechanically lysed bacterial cells produced AuNPs. In this case, the purple color was observed after 96 h of incubation at 42 °C (Fig. 3A). In both cases, peaks presumably corresponding to AuNPs were identified at 530 nm. Figure 3B shows HR-TEM images of the AuNPs synthesized using autoclave-dead and mechanically lysed *P. aeruginosa* cells. The AuNPs were spherical, triangular, and hexagonal and agglomerated on the bacteria’s surface. The average sizes of AuNPs synthesized using dead and lysed bacteria were 25.7 ± 5.3 nm and 53.0 ± 14.2, respectively. The lattice spacing of AuNPs was measured at 0.21 nm and 0.24 nm, corresponding to (200) and (111) planes of Au, indicating AuNPs were formed in the presence of dead and lysed bacteria. EDS analysis also demonstrated the presence of Au in the AuNPs.

### Evaluation of peptidoglycan and lipopolysaccharides for AuNP synthesis

Since inactivated bacteria could produce AuNPs, we next wanted to investigate whether cell wall components, such as peptidoglycan or lipopolysaccharides, could participate in the synthesis of nanoparticles. For this purpose, similar experiments were conducted using products (Fig. S2). A purple color appeared within 24 h (37 °C) after incubation of the gold salt with 2.0 mg/mL of *B. subtilis* peptidoglycan with 0.5 mM DS-AuCl_4_. The peak of AuNPs was observed at 530 nm, indicating the successful synthesis of AuNPs. However, lower concentrations did not display color change (Fig. S2Aa). In contrast, no AuNPs were synthesized in any *E. coli* lipopolysaccharide tested (Fig. S2Ab). Figure S2B shows HR-TEM images of the AuNPs synthesized using peptidoglycan. AuNPs were spherical, triangular, and hexagonal. The average size of AuNPs was 43.6 ± 11.3 nm. The measured lattice spacings of AuNPs were 0.12 nm and 0.24 nm, corresponding to the (222) and (200) planes of metallic Au. EDS analysis demonstrated the presence of Au in AuNPs (Fig. S3).

### Extracellular synthesis of AuNPs using P. aeruginosa metabolites

**Fig. 4:**
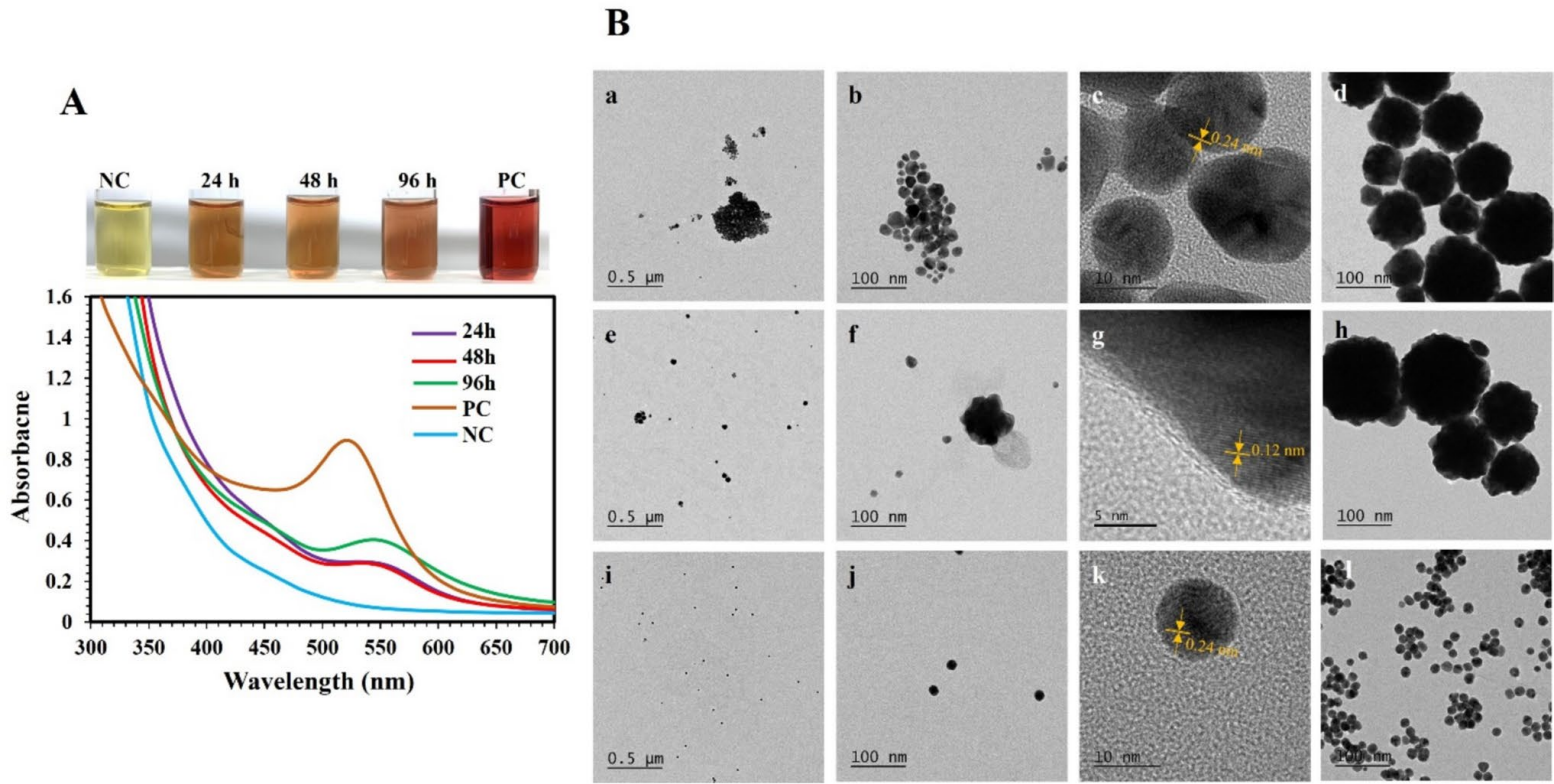
UV–vis and TEM investigation of AuNPs synthesized using *P. aeruginosa* extracellular metabolites. Extracellular metabolites produced after 24, 48, and 96 h of growth were incubated with 0.5 mM DS-AuCl_4_ at 42 °C. (**A**) UV–vis after 10 days of incubation at 42 °C and test tubes displaying the corresponding colors. NC is the negative control (nutrient broth), and PC is the positive control (NaBH_4_). (**B**) HR-TEM analysis of AuNPs synthesized using extracellular metabolites after 24 h (a–d) and 96 h of growth (e–h), and the positive control sodium borohydride (i–l). Images after 10 days of incubation at 42 °C (a–h). In the case of positive control, images were taken after 24 h of incubation. Lattice spacing of AuNPs is shown in orange. The experiments were repeated at least three times. Representative images of UV–vis, test tubes, and HR-TEM images are shown

Similar experiments were conducted to study the synthesis of AuNPs using *P. aeruginosa* extracellular metabolites using metabolites collected at different time points of growth (24, 48, and 96 h). Extracellular metabolites from the three-time points exhibited the capability to synthesize AuNPs, indicated by a brown color that developed within seven days. It was observed that the color intensity reached its maximum after ten days. In this case, AuNP peaks were observed at 550 nm (Fig. 4A). Among the conditions, the metabolites obtained after 96 h produced a slightly intense color and a higher peak. Regarding control samples, the negative control (nutrient broth) did not display color change. In contrast, the positive control (NaBH_4_) developed a red cherry color after 24 h of incubation with AuNPs peak at 530 nm (Fig. 4A).

Figure 4B shows HR-TEM images of the AuNPs synthesized using extracellular metabolites and the positive control. The morphologies of synthesized AuNPs using extracellular metabolites after 24 h of growth were spherical, flower-shaped, and agglomerated. The average size of AuNPs in bacterial metabolites after 24 h, 96 h, and sodium borohydride (control) was 18.4 ± 6.0 nm, 23.8 ± 13.4 nm, and 18.1 ± 2.0 nm, respectively. The lattice spacings of AuNPs were measured at 0.12 nm and 0.24 nm, corresponding to the (222) and (220) planes of Au. EDS analysis showed the presence of Au in the AuNPs (Fig. S4).

### Additional characterization of the AuNPs

The morphology and composition of synthesized AuNPs using bacteria were analyzed using HR-TEM and EDS. The morphologies of the AuNPs were spherical, triangular, star, and hexagonal (Fig. 1; Table 1). In general, AuNPs were aggregated on the surface of the bacteria. The size of the AuNPs was very diverse, depending on the synthesis conditions, including temperature, pH, living or inactivated bacteria, peptidoglycan, and metabolites. The lattice spacings of 0.12, 0.13, 0.21, 0.24, and 0.25 nm were measured using Fast Fourier transform (FFT) analysis with Image J. As reported in the literature, the lattice spacing of metallic Au is measured at d_111_ = 0.24 nm, d_222_ = 0.12 nm, d_220_ = 0.15 nm, d_020_ = 0.21 nm, d_311_ = 0.13 nm, d_131_ = 0.13 nm, and d_402_ = 0.09 nm (Bandyopadhyay et al. 2017). The lattice spacing analysis confirmed that the synthesis methods produced metallic AuNPs. EDS analysis confirmed the presence of gold in the AuNPs.

**Table 1:** Summary of the conditions used in the study and the shapes of the synthesized AuNPs

Conditions	Temp	pH	Reduction time	AuNPs morphology
25 °C (48 h)	25 °C	3.71	48 h	Sphere, triangle, hexagon
37 °C (24 h)	37 °C	3.71	24 h	Sphere, star, triangle, hexagon
42 °C (24 h)	42 °C	3.71	24 h	Sphere, triangle, truncated triangle
Autoclave-dead cells	42 °C	3.67	24 h	Sphere, star, triangle, hexagon
Mechanically lysed cells	42 °C	3.67	24 h	Sphere, star, triangle, hexagon
Extracellular metabolites (24 h)	42 °C	3.52	10 days	Sphere, flower-shape
Extracellular metabolites (96 h)	42 °C	3.52	10 days	Sphere, flower-shape
pH 3.7	42 °C	3.7	24 h	Sphere, star, triangle, hexagon, truncated triangle
pH 7.0	42 °C	7.0	24 h	Sphere
pH 12.7	42 °C	12.7	24 h	Sphere
Peptidoglycan	42 °C	3.15	24 h	Sphere, triangle, hexagon
LPS	42 °C	3.15	-	-

The morphology of AuNPs was analyzed using FE-SEM, Fig. S5. FE-SEM images demonstrated that the AuNPs were triangular and primarily spherical. Furthermore, AuNPs were aggregated onto the surface of bacteria used for the synthesis in this study. The results of the SEM analysis agreed with the HR-TEM results in showing typical morphologies of AuNPs.

Synthesized AuNPs were characterized further using X-ray powder diffraction (XRD) for phase identification of the crystalline material. The P-XRD patterns of AuNPs obtained under different experimental conditions are shown in Fig. S6. Major diffraction peaks at 2θ = 38.1 and 44.2 correspond to standard Bragg reflections for Au at (111) and (200) of the face-centered cubic lattices. The intensity of (220) and (211) are very low to detect, which might be due to the bacterial matrix covered on the surface or due to the growing preference for Au(0) orientation fixed in the (111) direction.

**Fig. 5:**
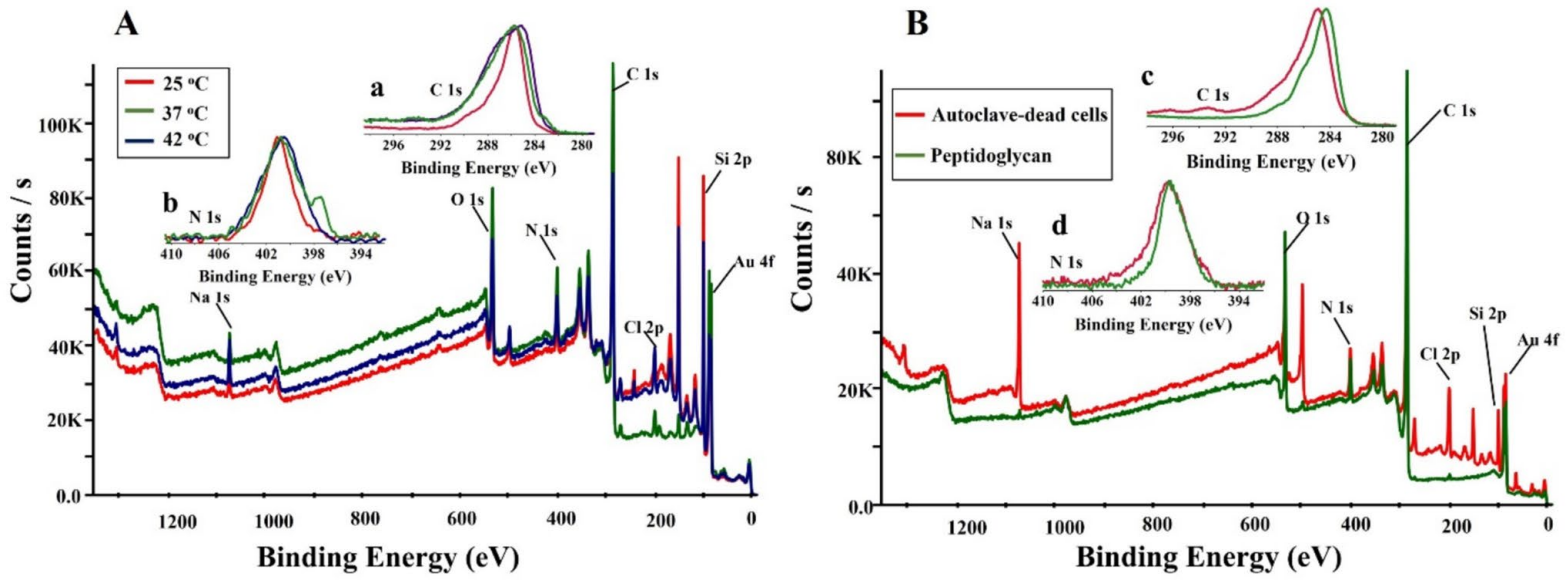
XPS survey spectra of AuNPs synthesized at different experimental conditions. (**A**) At different temperatures. (**B**) Using autoclave-dead bacterial cells and peptidoglycan. Inset shows C1s (a, c) and N1s narrow regions (b, d) of the respective synthesis strategies

**Fig. 6:**
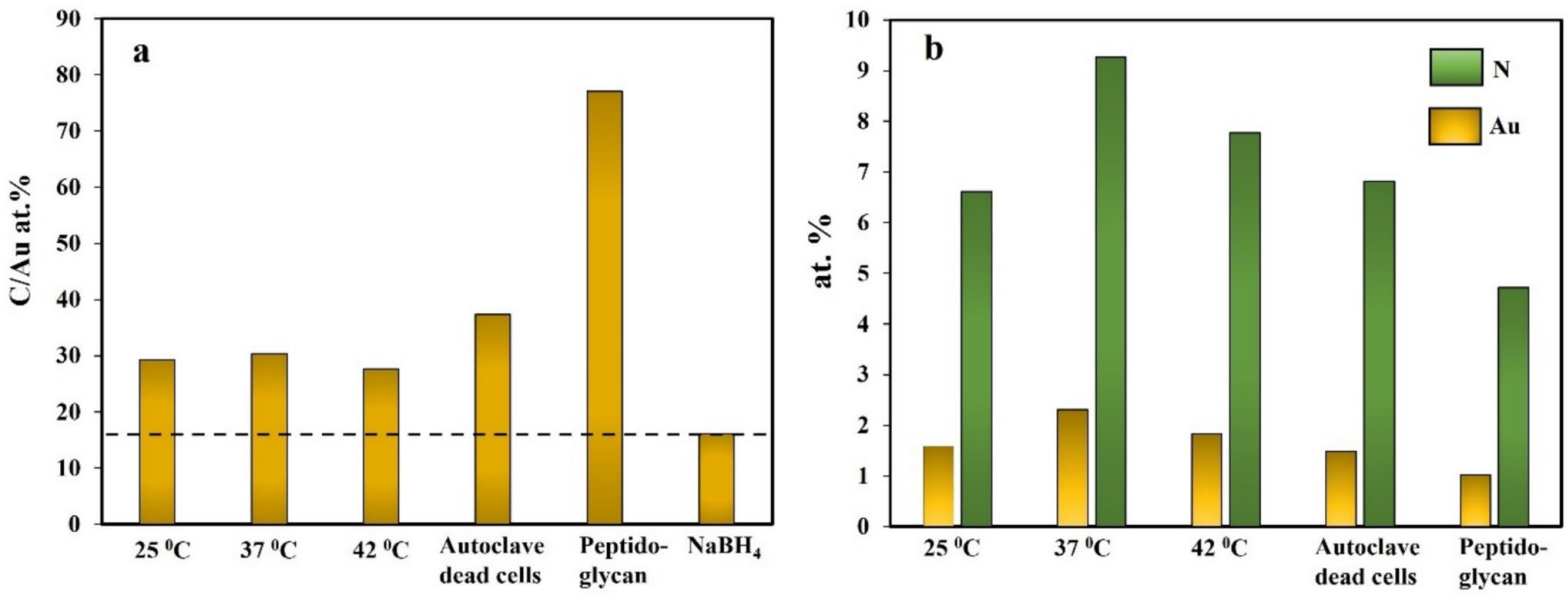
XPS surface analysis of AuNPs synthesized under different experimental conditions. Elemental composition C/Au at.% (**a**), N/Au at.% at various conditions (**b**), showing increased N presence originating from bacterial debris

The elemental composition of the AuNPs was investigated using X-ray photoelectron spectroscopy (XPS). Results were obtained by drop-casting the samples on a silicon wafer substrate and dried at room temperature. Figure 5A displays survey regions from AuNPs synthesized at different temperatures of 25 °C (48 h), 37 °C (24 h), and 42 °C (48 h), and Fig. 5B reveals survey regions of autoclave-dead bacteria and peptidoglycan-mediated synthesis. C1s, Au4f doublet, N1s, and O1s are centered at 285, 84–88, 400, and 532 eV. Figure 5a–d displays C1s and N1s narrow regions of bacterial reduced AuNPs at different temperatures. Figures 5a and 6c show the C1s region, which has three prominent peaks, C–C/C-H, C-O/C-N, and particularly N–C = O, arising from bacterial components. In contrast, a minor feature due to the COOH group from the aryl shell is present at 289.1 eV (Gam-Derouich et al. 2010). Figure 5b and d shows a narrow scan for N1s, and the peak is narrower and centered at 400.0 eV due to peptide links in the bacterial components.

Elemental composition in the atomic percentage of AuNPs is depicted in Fig. 6, showing quantitative XPS surface analysis of AuNPs. The C1s/Au4f intensity ratio increased sharply compared to chemically reduced AuNPs-COOH using NaBH_4_ (Fig. 6a) due to bacterial debris on the AuNPs surface. Another significant difference between the survey regions lies in the sharp N1s peaks from the bacterial bioconjugates compared to bare gold-aryl nanoparticles (0 at. wt%) due to peptide links of bacterial proteins (Fig. 6b).

### Biocompatibility of AuNPs synthesized using P. aeruginosa cells and extracellular metabolites

To determine the biocompatibility of AuNPs synthesized using *P. aeruginosa*, antibacterial activities, and hemocompatibility assays were performed on AuNPs synthesized using live bacterial cells and extracellular metabolites collected after 96 h of growth. The results of the antibacterial activity of AuNPs showed no inhibition against *E. coli* and *S. aureus*, supported by (1) the presence of turbidity, (2) the presence of a round bottom larger than 2 mm, and (3) the conversion of blue resazurin to pink resorufin after 2 h of incubation in the dark (Fig. S7). These observations indicate that AuNPs are nontoxic to these bacteria.

**Fig. 7:**
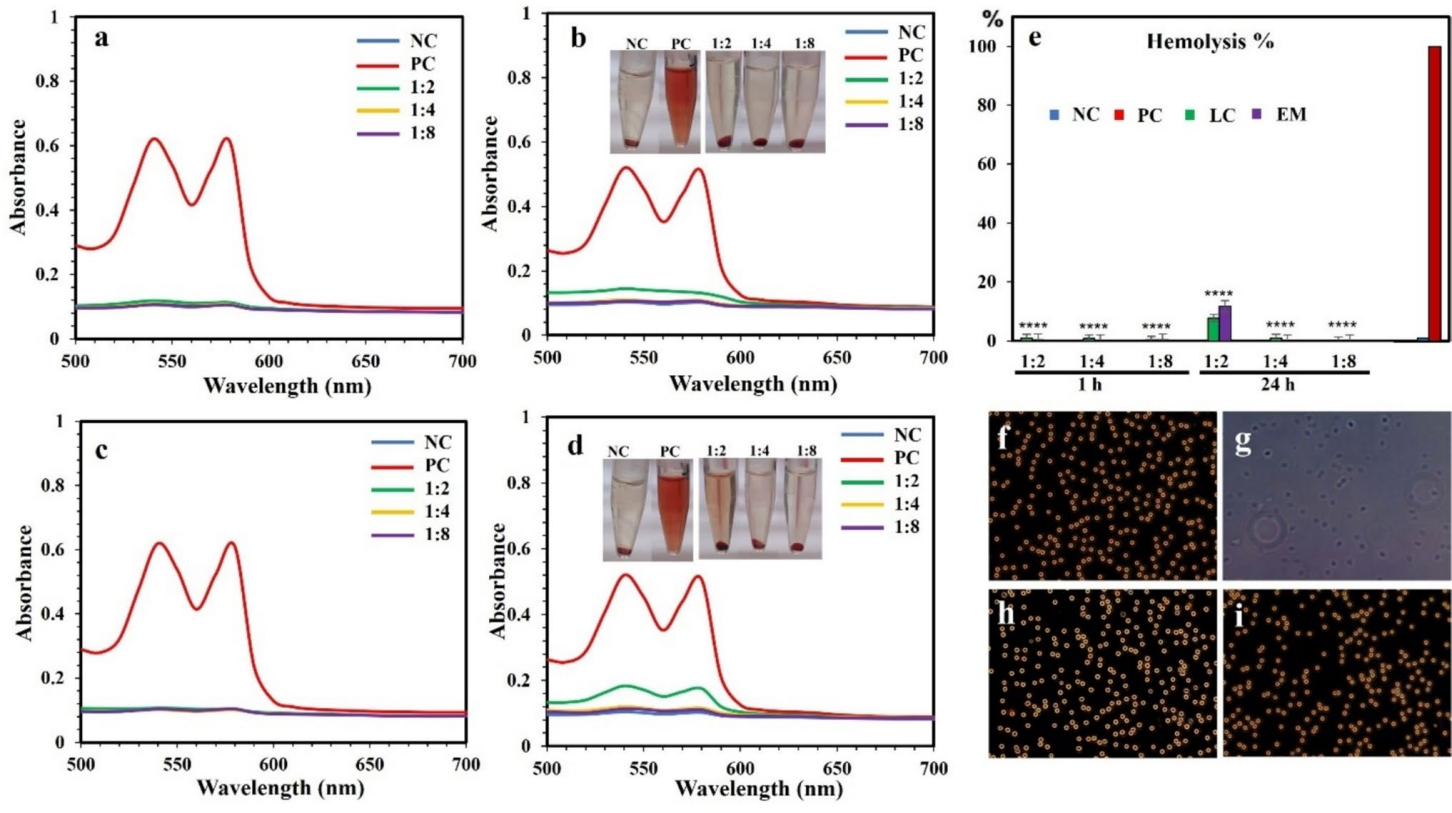
Hemolysis assay with human red blood cells using AuNPs from reactions with live cells and extracellular metabolites after 96 h growth. Effect of AuNPs synthesized using live bacterial cells after 1 h (**a**) and 24 h (**b**) incubation at 37 °C. Images of test tubes at 1:2, 1:4, and 1:8 dilutions after 24 h incubation. Effect of AuNPs synthesized using extracellular metabolites after 1 h (**c**) and 24 h (**d**) incubation. Images of test tubes at 1:2, 1:4, and 1:8 dilutions after 24 h incubation. (**e**) Percentage of hemolysis after 1 h and 24 h of incubation (**f**–**i**) Images of human red blood cells under phase contrast microscope after 1 h incubation corresponding to negative control (saline solution) (**f**), positive control (deionized water) (**g**), dilution 1:4 of AuNPs synthesized using live cells (**h**), dilution 1:4 of AuNPs synthesized using extracellular metabolites (**i**). Abbreviations are NC, negative control (saline solution); PC, positive control (deionized water); LC, live cells; EM, extracellular metabolites. The experiments were repeated at least three times, and representative images of UV–vis, test tubes, and RBCs images were shown. The hemolysis % data represented as mean values of the replicates (n3); crossbars represent ± standard errors, and samples statistically different from the control were marked with ****for *p*-values < 0.0001

The results of hemocompatibility tests of AuNPs synthesized using live bacterial cells and extracellular metabolites collected after 96 h of growth, Fig. 7. Results show no hemolysis after 1 h of incubation with 1:2, 1:4, and 1:8 dilutions of AuNPs. However, after 24 h of incubation, no hemolysis was observed at the lower dilutions of 1:4 and 1:8, whereas, at the higher dilution of 1:2 a slight insignificant hemolysis of 8% and 12% was observed for AuNPs synthesized using live cells and extracellular metabolite, respectively. In comparison, the positive control (RBCs in deionized water) exhibited 100% hemolysis. The morphological changes induced by AuNPs on human RBCs were studied under phase contrast microscopy, Fig. 7. RCBs displayed standard cell morphology and size, and a negligible number of hemolyzed cells of 1–2% was observed after 1 h of incubation (Fig. 7h–i). The negative control (saline solution) showed no hemolysis (Fig. 7f), whereas complete hemolysis was observed with the positive control (deionized water), showing ghost cells formation (Fig. 7g).

## Discussion

Biocompatible anisotropic AuNP synthesis using green methods has gained significant attraction due to the numerous favorable properties arising from anisotropy and the absence of harmful chemicals (Fatima et al. 2024; Kohout et al. 2018). Using microorganisms to synthesize nanoparticles is an exciting and emerging field (Mandal et al. 2006). In this study, *P. aeruginosa* was used to synthesize anisotropic AuNPs from DS-AuCl_4_ salt at a cell density of 6 × 10^8^ cells/mL under three temperatures of 25 °C (48 h), 37 °C (24 h), and 42 °C (24 h). Bacterial concentration was a crucial factor in AuNP synthesis, and higher temperatures of 37 °C (24 h) and 42 °C (24 h) speed up the formation of the nanoparticles. HR-TEM images showed that the synthesized AuNPs using *P. aeruginosa* were anisotropic, and the measured lattice spacing for all AuNPs indicates that bacteria synthesized AuNPs at different temperatures, as previously described (Han et al. 2014; Song et al. 2014). The results suggested that bacterial concentration plays a significant role in reducing DS-AuCl_4_ salt. These findings are consistent with previous studies. For example, in the marine yeast *Yarrowia lipolytica* NCIM 3589, the synthesis of AuNPs from HAuCl_4_ was affected by cell density (Pimprikar et al. 2009). Similarly, it was reported that the rate of AuNP formation using some bacterial and fungal strains was directly related to the incubation temperature (Gericke and Pinches 2006). They found that the higher the temperature, the faster the particle synthesis. Recently, similar results were reported in the AuNP synthesis using *Nocardiopsis dassonvillei* NCIM 5124 (Bennur et al. 2020). Other studies investigated the impact of temperatures on AuNP synthesis without biological agents. Thus, using poly dimethylaminoethyl methacrylate homopolymer without a reducing agent (Mountrichas et al. 2014) or laser ablation in gum Arabic (Mzwd et al. 2022), it was also concluded that high temperatures led to faster formation of AuNPs.

Additionally, we evaluated the effect of pH on the AuNP synthesis. Results showed that anisotropic AuNPs were synthesized at pH 3.7, while 7.0 and 12.7 AuNPs were spherical. A blue shift in the absorption spectra from 530 to 520 nm was observed. A similar blue shift was observed when AuNPs were conjugated with proteins, such as the AuNPs lysozyme bioconjugate, which exhibited a plasmon peak shift from 550 to 520 nm at pH 12.0. This shift was accompanied by the formation of nanoparticles with a uniform size of 18 ± 10 nm (Ahmady et al. 2019). Moreover, a broad absorption peak was observed at neutral pH, suggesting that the nanoparticles possess a range of dimensions or structural characteristics (Parambath et al. 2023a, b). A similar study reported that the synthesis of AuNPs using yeast was regulated at a pH of 4.5 (Pimprikar et al. 2009). Moreover, He et al. (2007) investigated the ability of *Rhodopseudomonas capsulate* bacteria to synthesize AuNPs at different pH values. They found that the pH affected the morphology and size of the AuNPs. At pH 4.0, anisotropic AuNPs were synthesized; however, at pH 7, spherical AuNPs were synthesized. The measured lattice spacing of AuNPs indicated that the formation of AuNPs was not affected by the pH (Han et al. 2014; Song et al. 2014). Based on these findings, it can be concluded that the pH affected the size and morphology of AuNPs.

Autoclave-dead and mechanically lysed *P. aeruginosa* cells have comparable efficiency to the live bacteria in reducing DS-AuCl_4_ and synthesizing anisotropic AuNPs. HR-TEM images showed that AuNPs were anisotropic, and the lattice spacing of AuNPs indicated that nanoparticles were effectively formed in inactivated bacteria. These results signify that the reduction of the DS-AuCl_4_ salt occurred using viable and nonviable bacteria, suggesting that the reduction does not require microorganism viability. In this line, researchers concluded that cell walls act as nucleation sites for metal nanoparticle synthesis (Park et al. 2016). A similar study investigated if the intact *E. coli* K12 was needed to synthesize AuNPs from HAuCl_4_. It was found that AuNP synthesis does not require intact cells (Srivastav et al. 2013). The synthesis of NPs was reported for dead bacteria with the same efficiency as live bacteria (Fang et al. 2019). Similar findings were reported in Hypocrea lixii fungi from copper (Salvadori et al. 2013) and *Aspergillus aculeatus* from nickel oxide (Salvadori et al. 2015).

Next, we tested whether significant components of the cell wall of Gram-negative bacteria, such as peptidoglycan and lipopolysaccharides, could synthesize AuNPs. When peptidoglycan was incubated with 0.5 mM DS-AuCl_4_, it synthesized AuNPs; however, lipopolysaccharides did not. HR-TEM images showed that the AuNPs synthesized using peptidoglycan were anisotropic. The measured lattice spacing of AuNPs confirmed that peptidoglycan was essential in reducing Au cations effectively (Bandyopadhyay et al. 2017; Zhou et al. 2013). Therefore, the cell wall, particularly the peptidoglycan, appears crucial in reducing the gold(III) salt to AuNPs. Peptidoglycan comprises N-acetylglucosamine and N-acetylmuramic acid layers, cross-linked by penicillin-binding proteins (Schaechter 2009). In comparison, lipopolysaccharides are glycoconjugates that contain a hydrophobic lipid domain (Lipid A) that binds to hydrophilic oligosaccharides (Farhana and Kha 2023). Typically, peptidoglycan contains D-Ala and D-Glu amino acids. Each amino acid has a carboxylic group, the primary site of metal deposition. This combination of biomolecules makes the peptidoglycan an ideal location for metal reduction. Additionally, Srivastava et al. (2013) confirmed that the synthesis of AuNPs using autoclave-dead *E. coli* K12 cells is controlled by extracellular membrane-bound proteins. Hence, proteins, peptides, and amino acids significantly reduce gold ions (Durán et al. 2011).

Extracellular metabolites produced using *P. aeruginosa* can synthesize AuNPs but at a slower rate than the bacterial cells. HR-TEM images and the lattice spacing of AuNPs confirmed that the bacterial metabolites could also reduce Au(III) to AuNPs (Bandyopadhyay et al. 2017; Zhou et al. 2013). A study by Husseiny et al. (2007) revealed that *P. aeruginosa* A TCC 90271 supernatants can synthesize 15–30 nm AuNPs. This could be due to the enzymes in *P. aeruginosa*, specifically nitrate reductase, which can reduce metals (Filiatrault et al. 2013). Fungi also contain nitrate reductase and thus can assist in fabricating metallic nanoparticles (Khandel and Shah 2016).

The P-XRD pattern indicates the anisotropy of the AuNPs, leading to the intense (111) peak relative to the other peaks for the assignments for Au, where the (111) and (200) plane diffraction peaks are located at 38.1 and 44.2, respectively (Parambath et al. 2023a, b). Spectral variations substantiated by precise quantitative X-ray photoelectron spectrometry (XPS) surface analysis show a sharp increase of C1s/Au4f intensity ratio compared to chemically reduced AuNPs-COOH using NaBH_4_ due to bacterial debris present on the NPs surface, which brings more nitrogen predominantly due to NH-C = O peptide links. Attenuating Au4f peaks and a concave up-shape arising from inelastic background scattering due to an underlying AuNP substrate were recently observed in several bioconjugates such as insulin-coated (AlBab et al. 2020) gold nanoparticles. Accordingly, the C1s region contained the three prominent peaks C–C/C-H, C-O/C-N, and particularly N–C = O arising from bacterial components. In contrast, a minimal feature due to the COOH group from the aryl shell is present at 289.1 eV (Gam-Derouich et al. 2010). Regarding the N1s region, the peak was narrower and centered at 400.0 eV, probably due to peptide links with bacterial components (Belsey et al. 2015).

The AuNPs synthesized using *P. aeruginosa* live bacteria and extracellular metabolites showed no inhibition and toxicity on *E. coli* and *S. aureus*, contrasting with the toxic DS-AuCl_4_ salt against *P. aeruginosa*. Furthermore, the results of the hemocompatibility assay of AuNPs showed no hemolysis after 1 h and negligible hemolysis at the high concentrations after 24 h. RBCs displayed normal cell morphology and size and an insignificant number of hemolyzed cells after 1 h of incubation, supported by Ahmady et al. 2019. Hence, the synthesized AuNPs using *P. aeruginosa* were hemocompatible with human RBCs.

In conclusion, this study demonstrated the successful green synthesis of anisotropic AuNPs using *P. aeruginosa* bacteria via the reduction of DS-AuCl_4_ salt. Several key factors, including bacterial concentration (6.0 × 10^8^ CFU/mL), temperature (42 °C), and pH (3.7), strongly affected the size and morphology of the synthesized AuNPs resulting in different shapes, including spheres, stars, triangles, hexagons, and truncated triangles. Cell wall components, such as peptidoglycan, were crucial in the DS-AuCl_4_ salt reduction. Inactivated bacterial cells produced more morphologies at a higher rate than bacterial metabolites. The average size of AuNPs synthesized at different temperatures was between 39.0 ± 9.1 nm and 26.0 ± 8.176 nm. Upon changing the pH, a change in the size was noticed, the average size became smaller at a pH of 12.7 (7.3 ± 2.5 nm). Meanwhile, TEM images of autoclave-dead and mechanically lysed bacterial cells showed average sizes of AuNPs 25.7 ± 5.3 nm and 53.0 ± 14.2, respectively. Evaluation of peptidoglycan TEM images of AuNPs were spherical, triangular, and hexagonal. The average size of AuNPs was 43.6 ± 11.3 nm. XRD showed intense diffraction peaks corresponding to Au(0) at (111) and (200). XPS analysis of AuNPs synthesized at different conditions displayed characteristic peaks for C1s, Au4f doublet, N1s, and O1s centered at 285, 84–88, 400, and 532 eV, respectively. The C1s region displayed three prominent peaks: C–C/C-H, C-O/C-N, and N–C = O, which are associated with bacterial components. Finally, the synthesized AuNPs were biocompatible and displayed no antibacterial activity. They are highly biocompatible with human RBCs, making them ideal drug carriers.

## Supplementary Information

Below is the link to the electronic supplementary material.

Supplementary file1 (PDF 1078 KB)

## Data Availability

All data supporting the findings of this study are available within the paper and its Supplementary Information.
