# Supplementary material for: Bacterial synthesis of anisotropic gold nanoparticles
Source: Appl Microbiol Biotechnol. 2025 Mar 10;109:62. doi: 10.1007/s00253-025-13438-w (PMC11893633; doi:10.1007/s00253-025-13438-w)
Supplement: Supplementary file 1 [file 253_2025_13438_MOESM1_ESM.pdf]

Bacterial synthesis of anisotropic gold nanoparticles

Islam M. Ahmady[1,2], Javad B. M. Parambath[3], Elsiddig A. E. Elsheikh[1], Gwangmin Kim[4], Changseok Han[4,5], Alejandro Pérez-García[2]*, Ahmed A. Mohamed[3]*

[1,2] Islam M. Ahmady	ORCID 0000-0003-0241-148X	(ialtayeb@sharjah.ac.ae)
[3] Javad B. M. Parambath	ORCID 0000-0002-5069-2143	(jbasil@sharjah.ac.ae)
[1] Elsiddig A. E. Elsheikh	ORICD 0000-0002-2949-5567	(eelsheikh@sharjah.ac.ae)
[4] Gwangmin Kim	ORCID 0000-0003-0653-222X	(rhkdals0731@gmail.com)
[4,5] Changseok Han	ORCID 0000-0002-8636-2859	(hanck@inha.ac.kr)
[2] Alejandro Pérez García	ORCID 0000-0002-1065-0360	(aperez@uma.es)
[3] Ahmed A. Mohamed	ORCID 0000-0001-7369-3117	(amohamed61@gmail.com)

[1]Department of Applied Biology, College of Sciences, University of Sharjah, Sharjah 27272, United Arab Emirates
[2]Departamento de Microbiología, Universidad de Málaga, and Instituto de Hortofruticultura Subtropical y Mediterránea "La Mayora" (IHSM-UMA-CSIC), 29071 Málaga, Spain
[3]Center for Advanced Materials Research, Research Institute of Sciences and Engineering, University of Sharjah, Sharjah 27272, United Arab Emirates
[4]Program in Environmental and Polymer Engineering, Graduate School of INHA University, 100 Inha-ro, Michuhol-gu, Incheon 22212, Korea
[5]Department of Environmental Engineering, INHA University, 100 Inha-ro, Michuhol-gu, Incheon 22212, Korea

*Co-corresponding authors

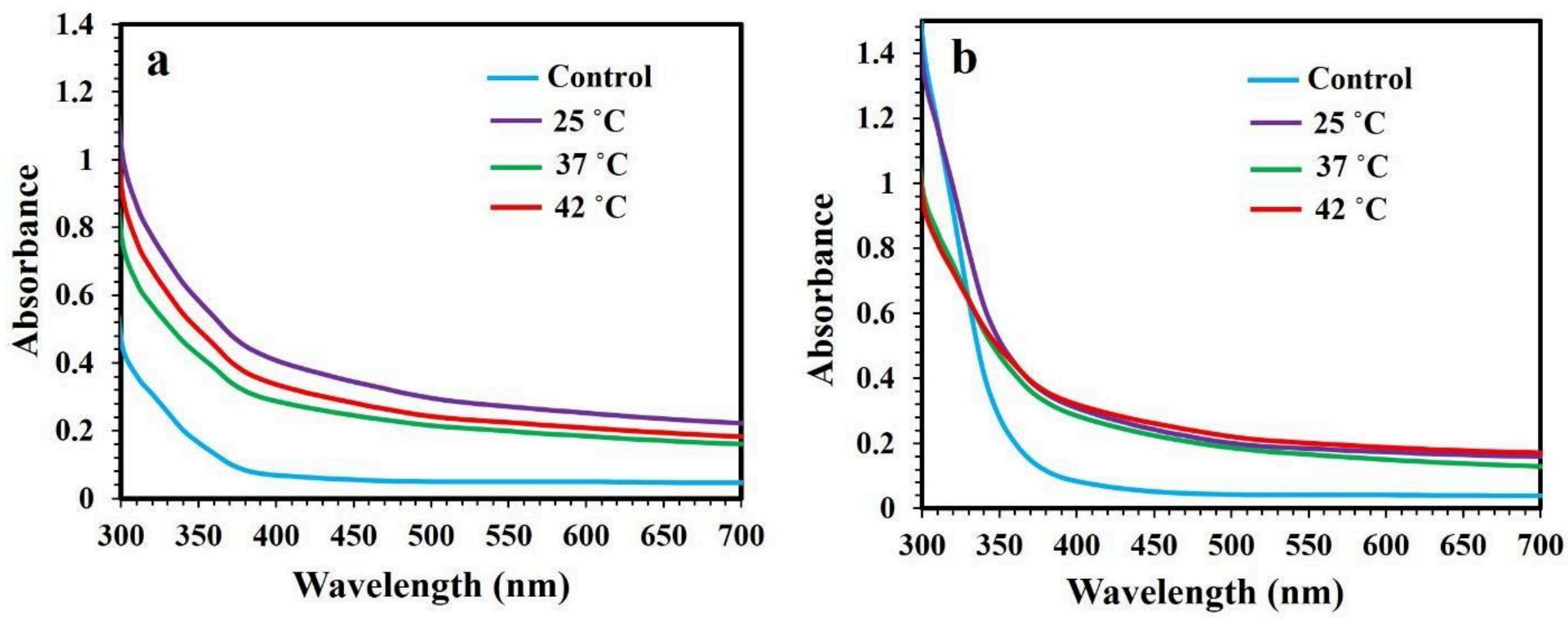

FIGURE S1 UV-vis investigation of attempt synthesis of AuNPs using *P. aeruginosa* ATCC 27853. UV-vis recorded after 48 h of incubation corresponding to bacterial cell suspensions of 1.5×10^8 CFU/mL (a) and 3×10^8 CFU/mL (b), incubated with 0.5 mM of DS-$AuCl_4$ at 25 ºC, 37 ºC, and 42 ºC. No nanoparticle peak was observed.

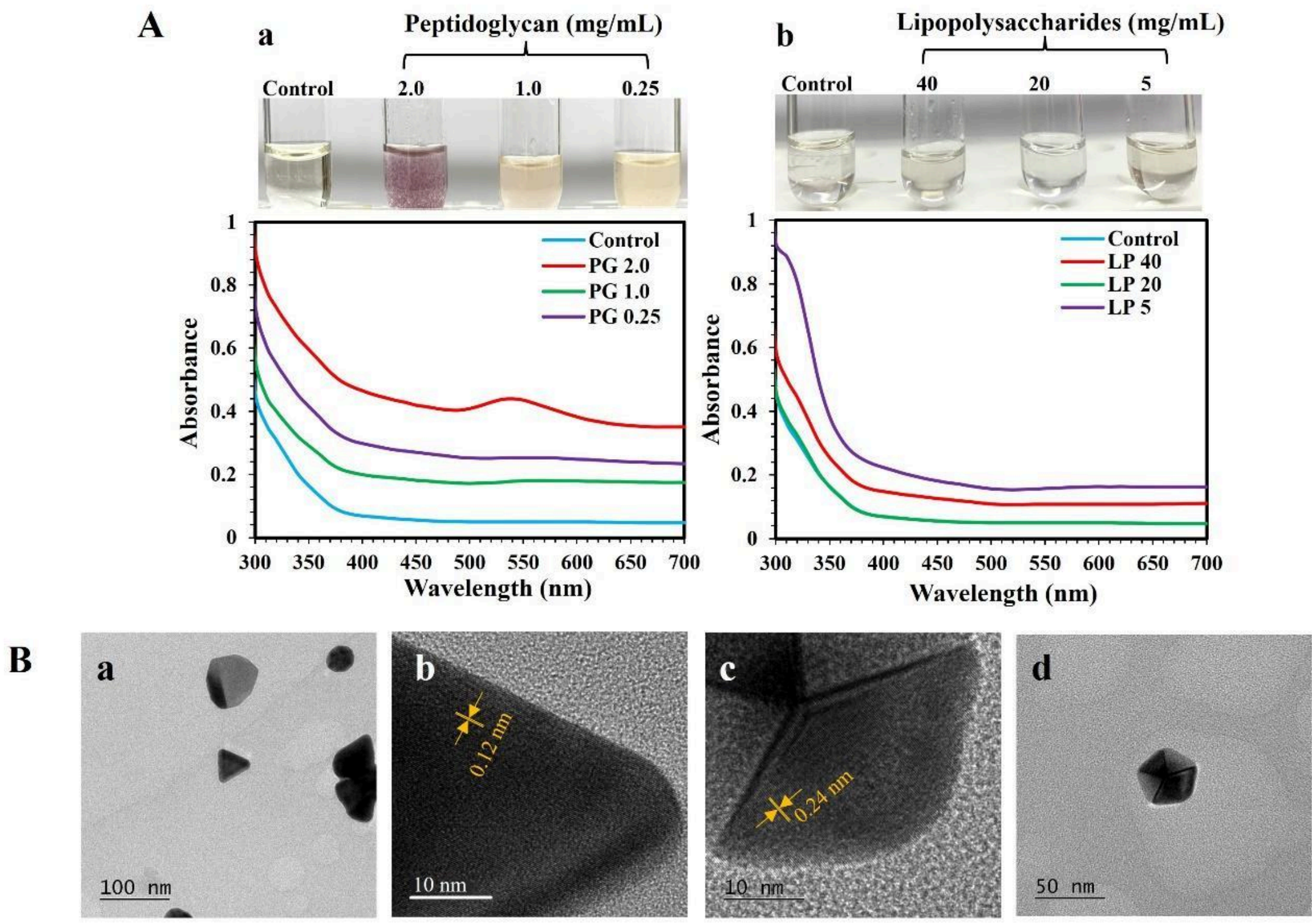

FIGURE S2 UV-vis and TEM investigation of AuNPs synthesized by peptidoglycan and lipopolysaccharides. (A) UV-vis of AuNPs synthesized using different concentrations of *B. subtilis* peptidoglycan (a) and *E. coli* lipopolysaccharides (b) incubated with 0.5 mM of DS-$AuCl_4$ at 42 ºC (24) h of incubation, and the corresponding test tubes. (B) HR-TEM analysis of synthesized AuNPs using *B. subtilis* peptidoglycan (a-d). Images were taken after one week of incubation at 42 ºC. The measured lattice spacing for some AuNPs is shown in orange.

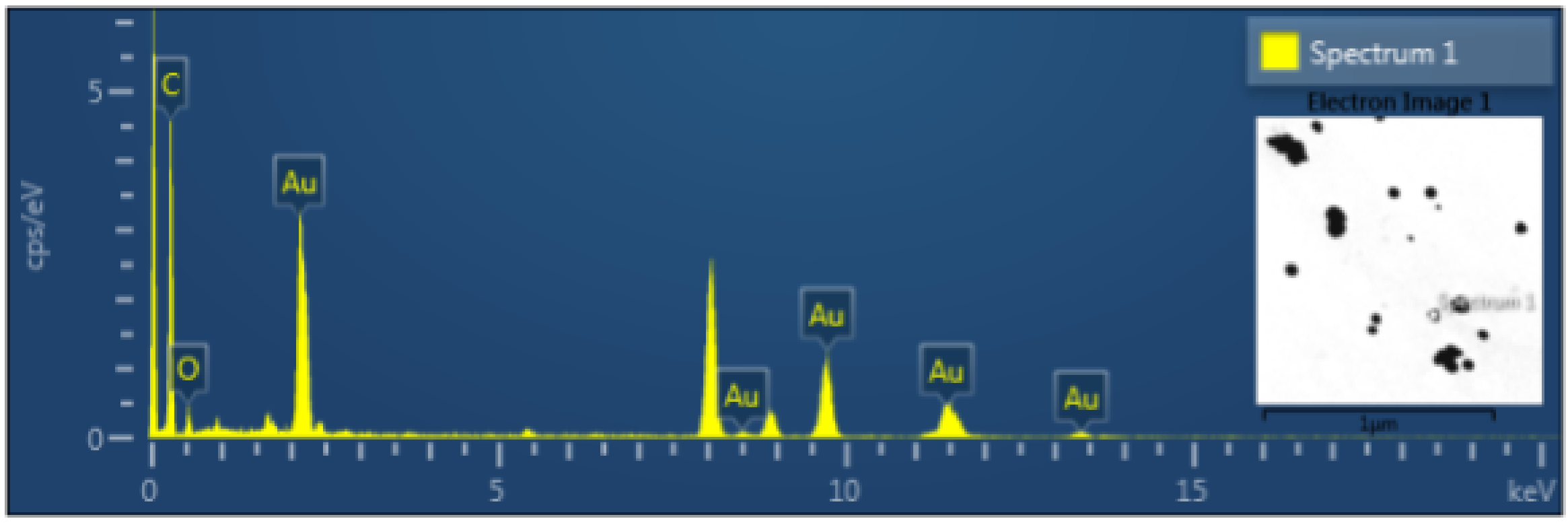

FIGURE S3 EDS of AuNPs synthesized using peptidoglycan. EDS was recorded after 2 mg/mL of *Bacillus* peptidoglycan incubated with 0.5 mM of DS-$AuCl_4$ at 37 °C (24 h).

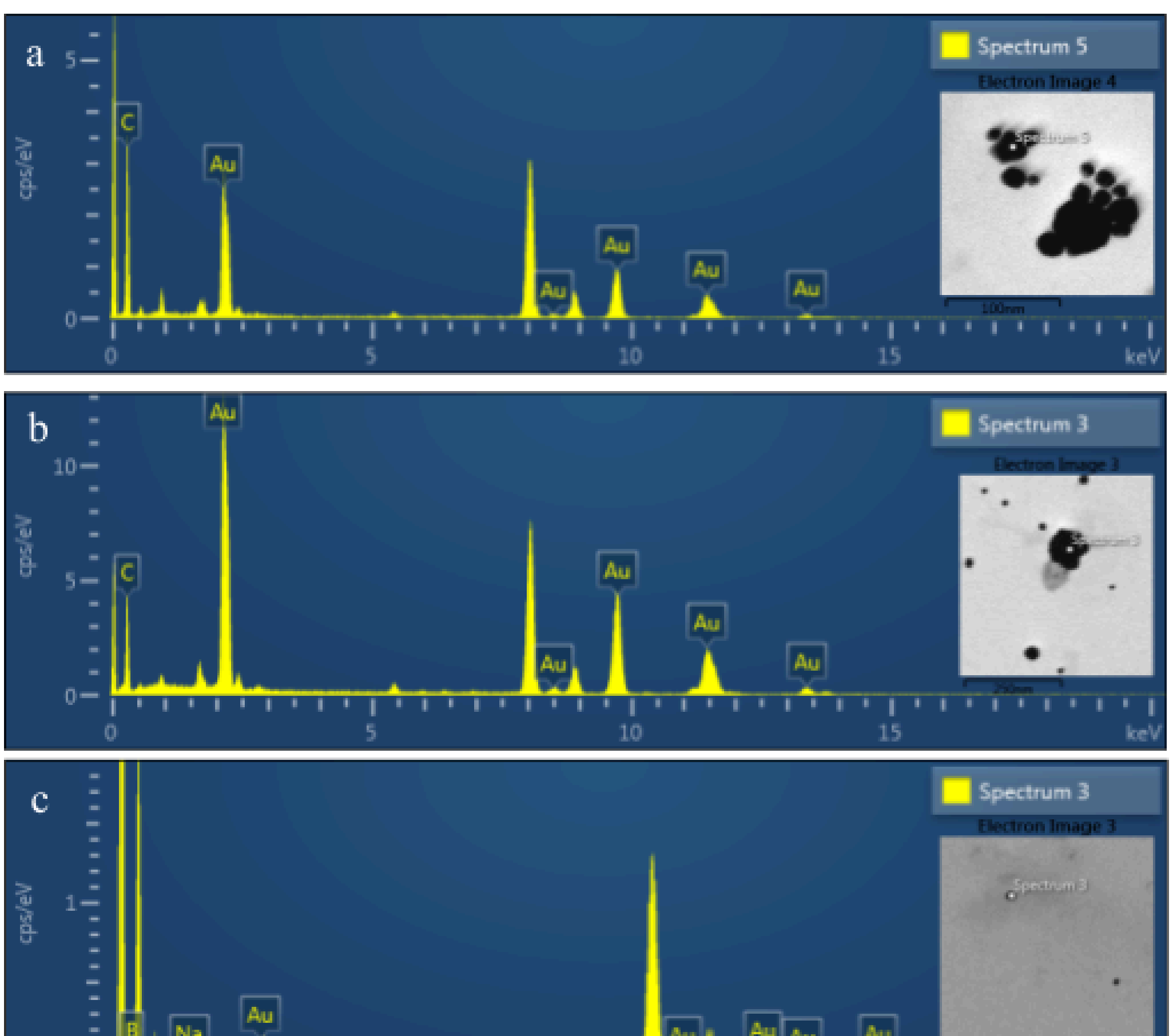

FIGURE S4 EDS analysis of AuNPs synthesized using extracellular *P. aeruginosa* ATCC 27853 metabolites. EDS after incubation of extracellular metabolites of 24 h (a) and 96 h (b) of growth with 0.5 mM DS-$AuCl_4$ at 42 ^{o}C. Sodium borohydride (c) was included as positive control.

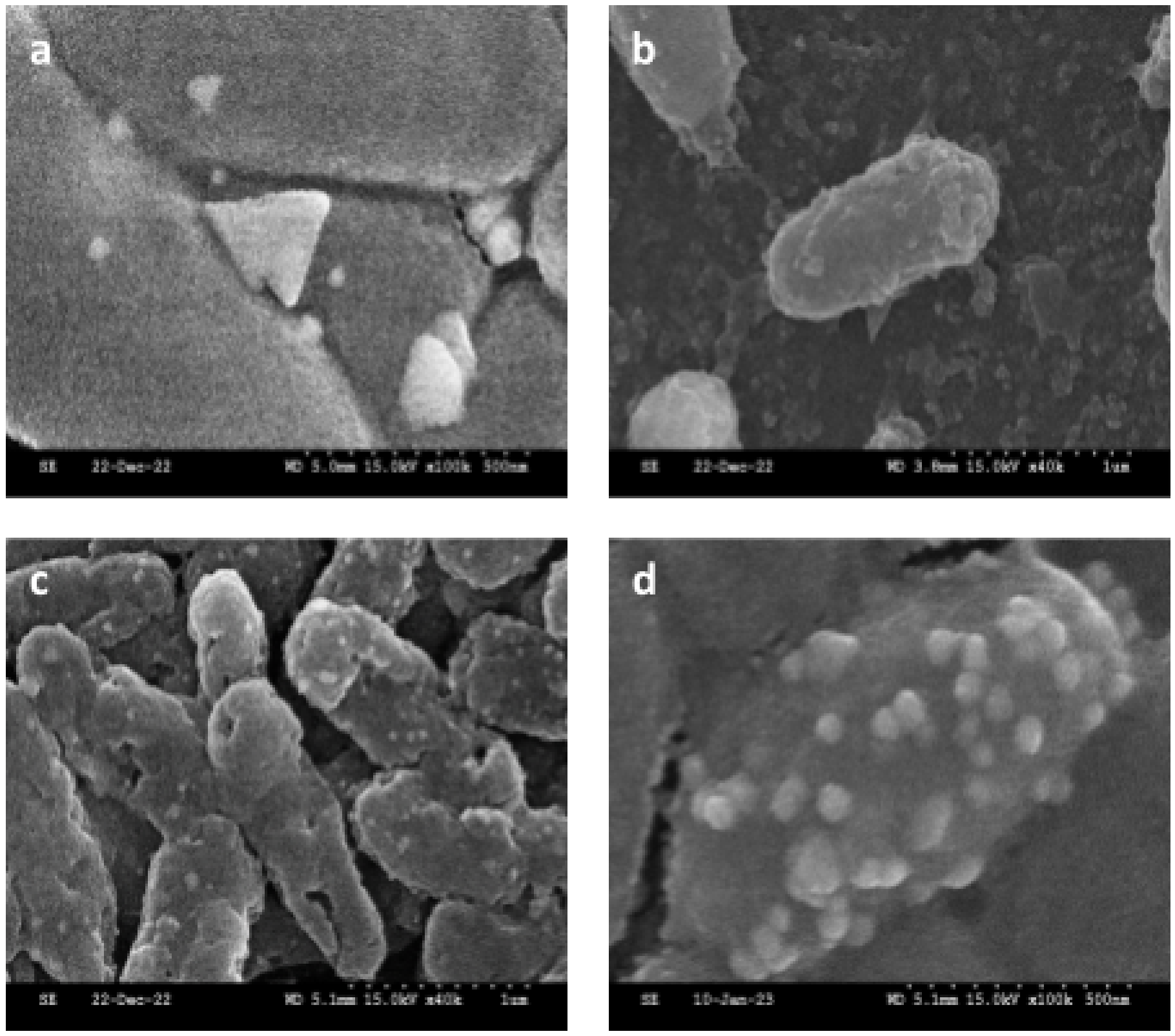

FIGURE S5 FE-SEM analysis of AuNPs synthesized using *P. aerug*inosa ATCC 27853. Images were taken from AuNPs obtained under different experimental conditions. After 24 h (42 °C) (a), at pH 12.7 (b), by autoclave-dead bacteria (c), and by mechanically lysed bacteria (d).

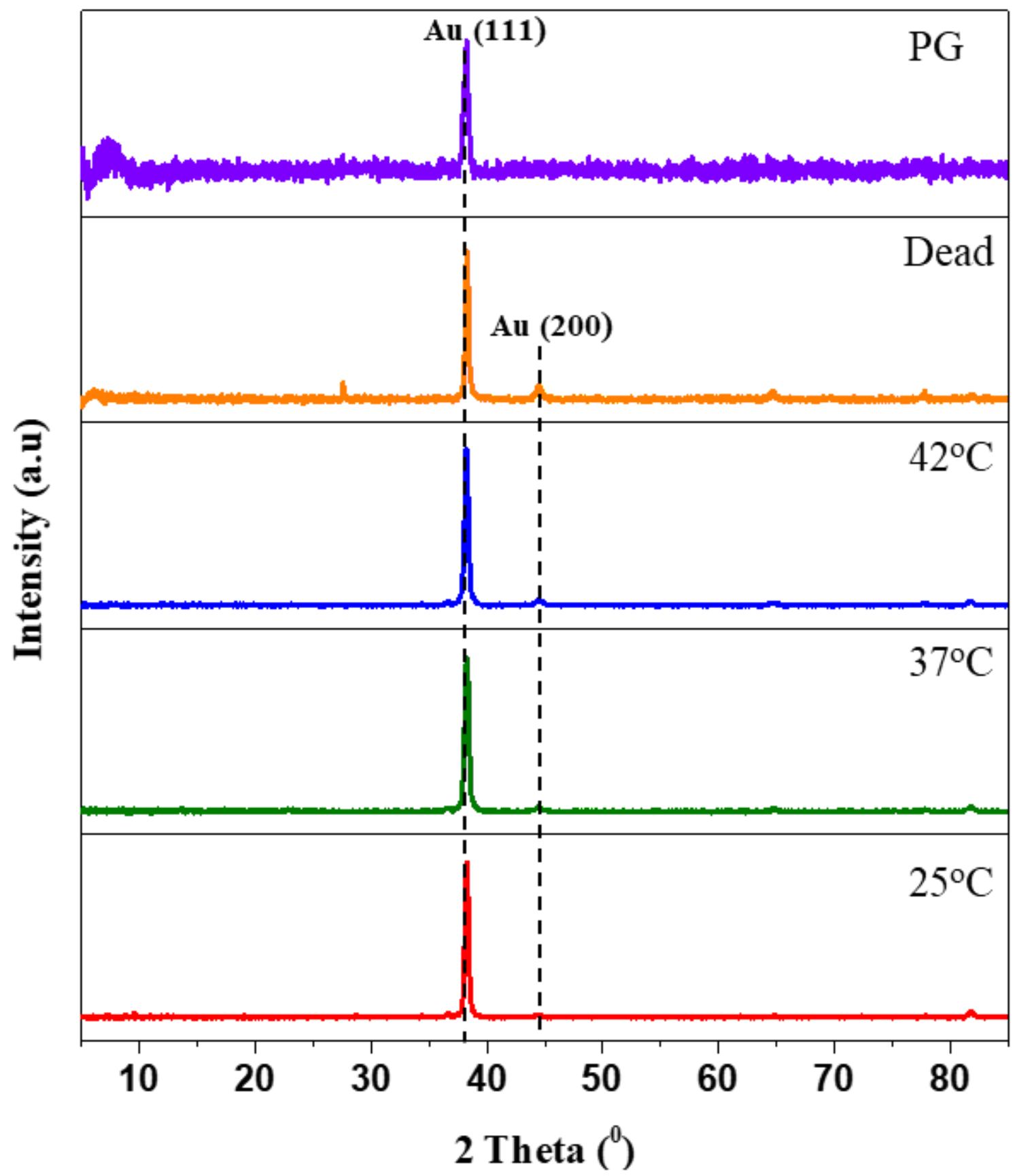

FIGURE S6 XRD of AuNPs obtained under different experimental conditions. P-XRD diffractograms of the AuNPs synthesized at 25 °C (48 h), 37 °C (24 h), and 42 °C (24 h), using autoclave-dead bacteria and *Bacillus* peptidoglycan (PG).

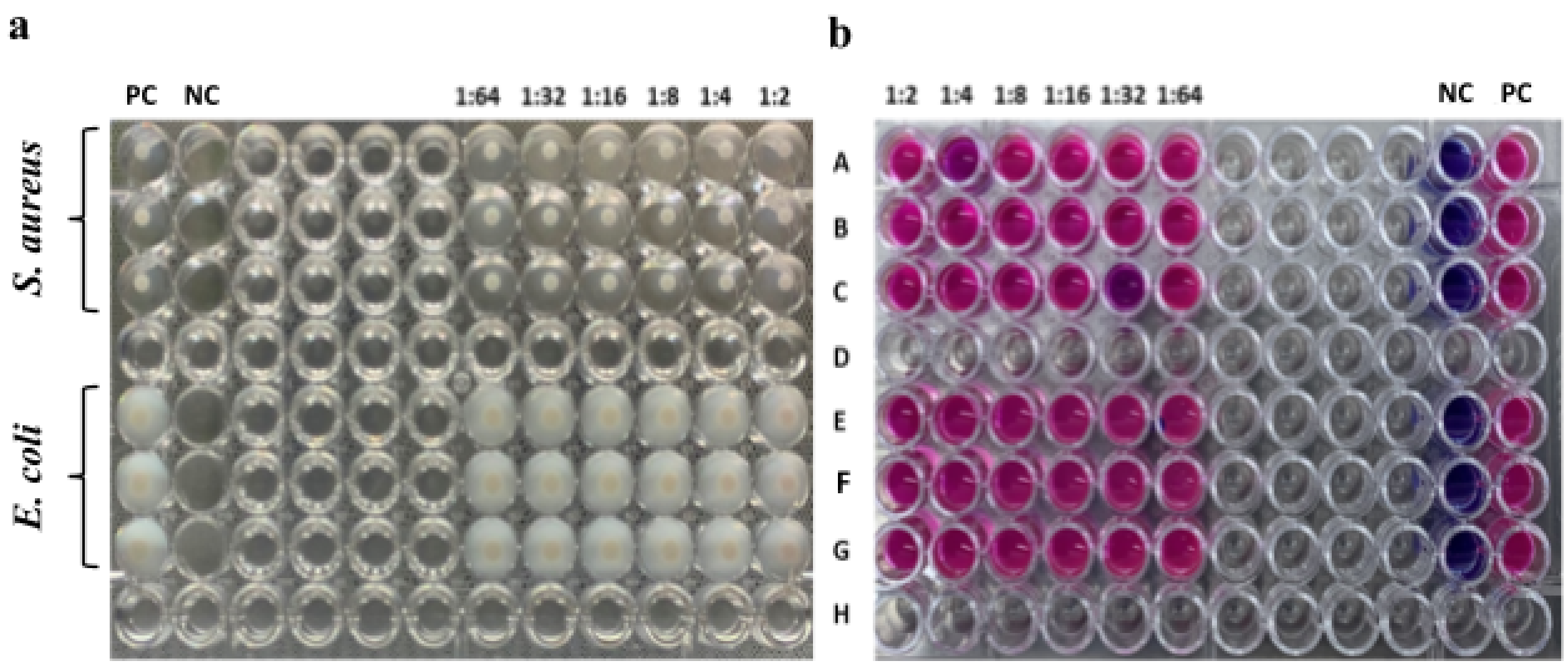

FIGURE S7. Antibacterial activities of AuNPs against *S. aureus* and *E. coli* using microplate assays. AuNPs produced by live cells were diluted twofold from 1:2 to 1:64 in Muller Hinton broth. (a) A 96-well plate showing bottom and turbidity, indicating growth for both bacterial species. (b) A 96-well plate treated with resazurin showing a reduction from blue to purple, indicating active bacterial growth in both cases. Bacteria were tested in triplicates: *S. aureus* was tested from rows A to C, and *E. coli* from rows E to G. PC, positive control with bacteria and no AuNPs; NC, negative control without bacteria. Similar results were observed when AuNPs produced using extracellular metabolites were tested (data not shown).
